# International variation in the management of severe COVID-19 patients

**DOI:** 10.1186/s13054-020-03194-w

**Published:** 2020-08-05

**Authors:** Elie Azoulay, Jan de Waele, Ricard Ferrer, Thomas Staudinger, Marta Borkowska, Pedro Povoa, Katerina Iliopoulou, Antonio Artigas, Stefan J. Schaller, Manu Shankar-Hari, Mariangela Pellegrini, Michael Darmon, Jozef Kesecioglu, Maurizio Cecconi

**Affiliations:** 1 Médecine Intensive et Réanimation, Department of the St-Louis Hospital, APHP, Hôpital Saint-Louis, Paris University, 1 avenue Claude Vellefaux, 75010 Paris, France; 2 Department of Critical Care Medicine, Ghent University Hospital, 9000 Gent, Netherlands; 3 Shock, Organ Dysfunction, and Resuscitation Research Group (SODIR), Instituto de Investigación de Vall d’Hebron, Barcelona, Spain; 4 Departmento de Medicina Intensiva, Hospital Universitario de Vall d́Hebron, Centro de Investigación Biomédica en Red (CIBER) de Enfermedades Respiratorias, Barcelona, Spain; 5 Department of Medicine I, Intensive Care Unit, Medical University of Vienna, Vienna General Hospital, Vienna, Austria; 6 NOVA Medical School, CHRC, New University of Lisbon, Unidade de Cuidados Intensivos Polivalente, Hospital de São Francisco Xavier, CHLO, Estrada Do Forte Do Alto Do Duque, 1449-005 Lisbon, Portugal; 7 Hellenic Army, ICU Nurse Manager General Military Hospital, Athens, Greece; 8 Critical Care Center, Sabadell Hospital, University Institute Parc Taulí, Autonomous University of Barcelona, Ciberes, Barcelona, Spain; 9 Charité - Universitätsmedizin Berlin, corporate member of Freie Universität Berlin, Humboldt-Universität zu Berlin, and Berlin Institute of Health, Department of Anesthesiology and Surgical Intensive Care, Berlin, Germany; 10 School of Immunology and Microbial Science, Kings College London. Guy’s and St Thomas’ NHS Foundation Trust, ICU Support Offices, St Thomas’ Hospital, London, UK; 11 Department of Surgical Sciences and Central Intensive Care Unit, Institute of Clinical Sciences, Sahlgrenska Academy, University of Gothenburg, Gothenburg, Sweden; 12 Department of Anesthesia, Operation, and Intensive Care; Institute of Clinical Sciences, Sahlgrenska Academy, University of Gothenburg, Gothenburg, Sweden; 13 Department of Anesthesiology and Intensive Care Medicine, Institute of Clinical Sciences, Sahlgrenska Academy, University of Gothenburg, Gothenburg, Sweden; 14 Department of Intensive Care Medicine, Division of Anesthesiology, Intensive Care and Emergency Medicine, University Medical Center Utrecht, Utrecht University, Utrecht, The Netherlands; 15 Humanitas Clinical and Research Center, Humanitas University, Milan, Italy

**Keywords:** Coronavirus, Acute respiratory distress syndrome, Viral infection, Remdesivir

## Abstract

**Background:**

There is little evidence to support the management of severe COVID-19 patients.

**Methods:**

To document this variation in practices, we performed an online survey (April 30–May 25, 2020) on behalf of the European Society of Intensive Care Medicine (ESICM). A case vignette was sent to ESICM members. Questions investigated practices for a previously healthy 39-year-old patient presenting with severe hypoxemia from COVID-19 infection.

**Results:**

A total of 1132 ICU specialists (response rate 20%) from 85 countries (12 regions) responded to the survey. The survey provides information on the heterogeneity in patient’s management, more particularly regarding the timing of ICU admission, the first line oxygenation strategy, optimization of management, and ventilatory settings in case of refractory hypoxemia. Practices related to antibacterial, antiviral, and anti-inflammatory therapies are also investigated.

**Conclusions:**

There are important practice variations in the management of severe COVID-19 patients, including differences at regional and individual levels. Large outcome studies based on multinational registries are warranted.

## Background

There is little evidence to support the optimal management of severe COVID-19 patients [1, 2]. To document whether there is a variation in practices, we performed an online survey (April 30–May 25, 2020) on behalf of ESICM.

## Methods

In this online survey, a case vignette (https://www.surveymonkey.com/r/F2FFC6S) was sent to ICU specialists who are members of ESICM. Questions investigated practices for a previously healthy 39-year-old patient presenting with severe hypoxemia from COVID-19 infection (Table 1). The 85 participating countries were grouped into 12 different regions [3]: continuous variables are described as median (interquartile range [IQR]) and are compared between groups using the non-parametric Wilcoxon rank-sum test. Categorical variables are described as frequency (percentages) and are compared between groups using Fisher’s exact test. Statistical analyses were performed with R statistical software, version 3.4.3 (available online at http://www.r-project.org/). A *p* value < 0.05 was considered significant.

**Table 1:** Distribution of the responses to the case vignette

Numbers (%) or median (interquartile ranges)	Total, 1001 respondents
**1. Admission to the ICU of a previously healthy 39-year-old man with severe COVID-19**	
Direct admission to the ICU	55%
Admission in an intermediate care unit	34.2%
Delayed admission to the ICU because of lack of bed	1.3%
Patient stays in the emergency department	0.8%
Patient admitted in the wards	8.8%
**2. Initial oxygenation strategy**	
I increase the oxygen flow to 15 l/min keeping the face mask	24.2%
I change the mask for a Venturi mask	17.5%
I start CPAP or noninvasive ventilation	25.5%
I start high flow nasal oxygen	47.1%
I intubate the patient right away	7.4%
I add prone positioning on spontaneous breathing	37.9%
**3. Optimizing oxygenation in a patient with a PF ratio of 84 4 h after intubation**	
I will give neuromuscular blockade for 24–48 h	50.9%
I increase and titrate PEEP to optimize recruitment	61.4%
I prone the patient immediately	73.2%
I am considering ECMO immediately	4.7%
Let us wait a little bit	9.9%
**4. Regarding the initial antibiotics**	
All my patients receive a broad anti-bacterial agent	45.3%
I only give broad anti-bacterial agent to febrile patients	11%
I only give broad anti-bacterial agent if CRP or PCT are high	4.2%
I only give broad anti-bacterials to patients with structural lung diseases	35.8%
I never give broad anti-bacterial agent in severe viral infections	3.7%
**5. Regarding initial anti-viral therapy, several options are possible**	
The level of evidence is so low that there is nothing I can give	48.9%
I prescribe (hydroxy)chloroquine	42.7%
I prescribe lopinavir/ritonavir	17.0%
I prescribe remdesivir	15%
I prescribe another anti-viral drug	4.6%
**6. Are you starting an anti-inflammatory therapy?**	
No	52.4%
Yes IL-1 or IL-6 blockade	24.8%
Yes, complement blockade	1.4%
Yes, steroids	31.5%
Yes, another anti-inflammatory drug	2.4%

## Results

Response rate was 20% (*N* = 1132 intensive care (ICU) specialists from 85 countries, including 1001 complete answers). Respondents (median 45 years [IQR, 39–53], 34% women) were from Middle Europe (25%), South Europe (23%), the United Kingdom (UK) (12%), South America (9%), North Europe (8.1%), Eastern Europe (5.3%), Middle-East (5%), North America (4.7%), Asia (3.3%), India (2.7%), Australia-New Zealand (1.3%), or Africa (0.6%); 54% were living in a large city (> 1 million inhabitants), and 55% were working in university-affiliated hospitals. The median (IQR) number of ICU beds was increased from 20 (11–36) to 35 (20–60) during the pandemic surge.

**Fig. 1:**
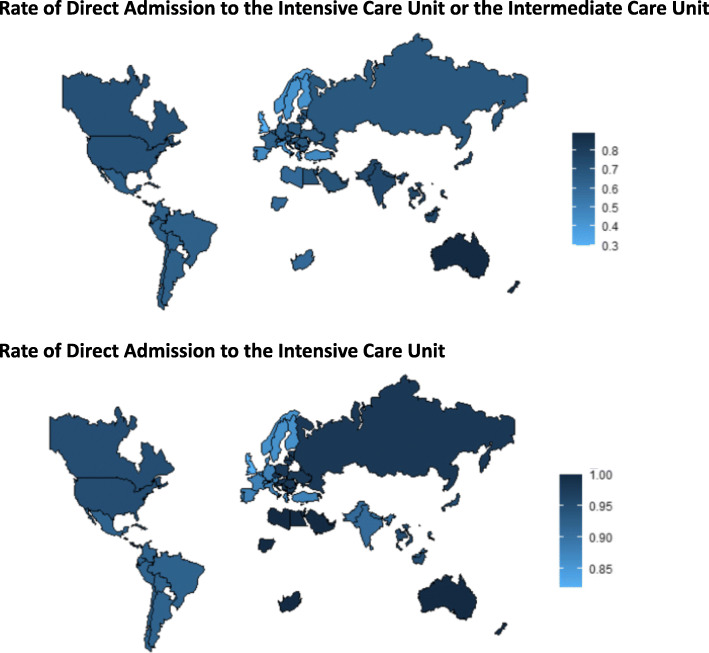
World map displaying practice variations across regions regarding direct admission to the ICU or the intermediate care unit

As the patient had 88 (peripheral oxygen saturation) SpO_2_ on 9 l/min of oxygen, direct ICU admission was reported in 56% (30–90%) of the respondents, with significant variation across regions (Fig. 1, *P* < 0.0001). Most intensivists not directly admitting patients to the ICU would admit them to an intermediate care unit managed by intensivists. However, the issue of bed availability was reported in South Europe (4.5%), South America (2.9%), Scandinavia (1.6%), Middle Europe (1.6%), and the UK (1.1%). Should the patient be not admitted to the ICU, a rapid response team would be involved in 29% of the cases, the ICU specialist would make the outreach her/himself in 24% of the cases, or an ICU nurse would be involved in 7% of the cases. In all other cases, ward or ED physicians would manage the patients. Direct ICU admission was significantly associated with baseline number of ICU beds (22 [12–40] vs. 18 [10–30] beds, *P* < 0.0001) and with the number of COVID-19 patients managed (30 [11–52] vs. 38 [20–70] patients, *P* = 0.001), as well as with management of patients in large cities (56.9% vs. 49.6%, *P* = 0.04).

**Fig. 2:**
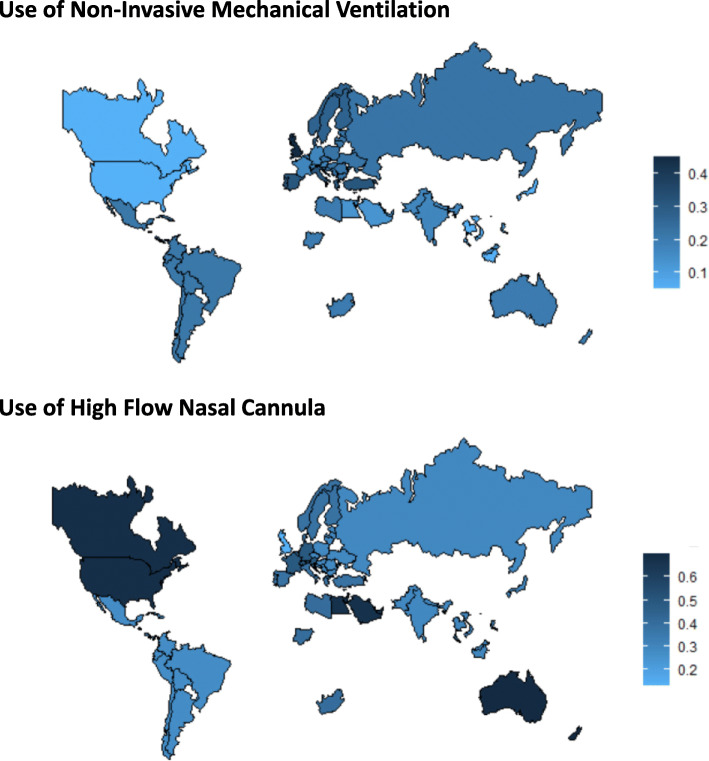
World map displaying practice variations across regions regarding the use of noninvasive oxygenation strategies

Respondents were then asked about the first-line oxygenation strategy, which varied significantly across regions (Fig. 2, *P* < 0.0001). First-line high flow nasal cannula (HFNC) was used by 22.9% of the respondents (0% in Australia-New Zealand, 38% in Eastern Europe). Noninvasive ventilation was used by 25.5% of the respondents (5.4% in North America, 43.6% in the UK). Interestingly, 8% of the respondents were using first-line intubation (0% in Australia-New Zealand, 23% in Asia). Women less frequently initiated HFNC (32% vs. 42%, *P* = 0.02). The availability of an intermediate care unit influenced the use of HFNC or non-invasive ventilation (NIV) (32.8% vs. 21.7%, *P* = 0.03). Along this line, a higher number of ICU beds (24 (12–40) vs. 18 (10–30) beds, *P* = 0.0009) was associated with the use of HFNC and NIV. Interestingly, 37.5% were using prone positioning in awake non-ventilated patients. To assess whether HFNC or NIV should be continued, ICU specialists relied on SpO_2_ (85.7%), respiratory rate (71.4%), followed by dyspnea (47.1%), and comfort (45.4%). Criteria for intubation included clinical signs of respiratory distress (94%), high oxygen flow to maintain a SpO_2_ of 95% (33.5%), or low SpO_2_ only (25.6%).

**Fig. 3:**
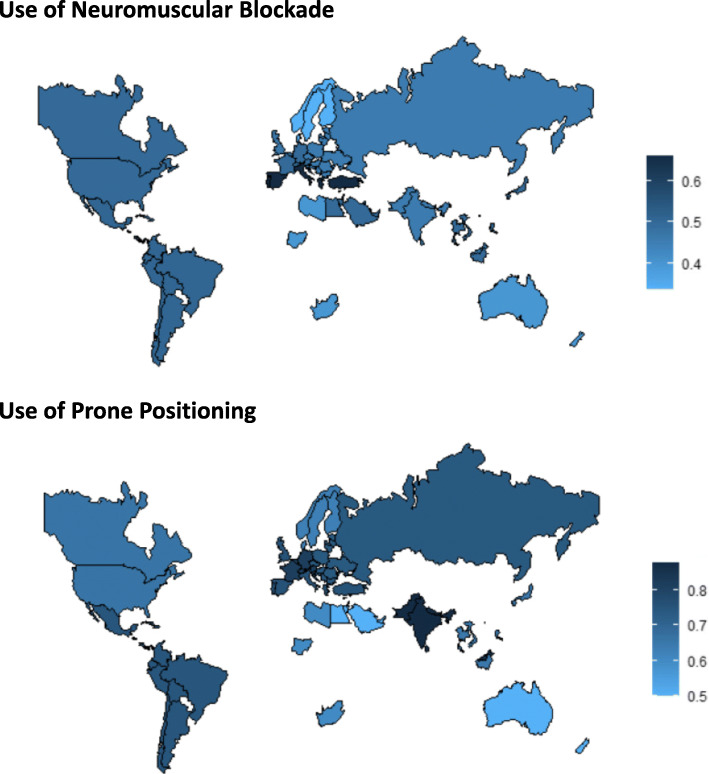
World map displaying practice variations across regions regarding the use of neuromuscular blockades and prone positioning

Following intubation, the patient had a partial pressure of oxygen/fraction of inspired oxygen (P/F) ratio of 84 mmHg. Although prone positioning (71.2%) and neuromuscular blockade (59.7%) were often used to optimize oxygenation, the practice varied significantly across countries. For instance, prone positioning was performed in 70–85% of the cases in Asia, India, Eastern Europe, Middle Europe, South America, South Europe, and the UK, whereas Africa, Australia-New Zealand, Middle East, North America, and Scandinavia were in the 50–70% range (Fig. 3, *P* < 0.0001). Respondent’s age was associated with the use of prone positioning (46 [39–54] vs. 44 [37–51] years, *P* = 0.007). Older respondent’s age (45 [37–52] vs. 47 [40–55] years, *P* = 0.0001), living in a large city (54.2% vs. 46.8%, *P* = 0.03), and a higher number of COVID-19 patients managed (35 [15–65] vs. 30 [12–55] patients, *P* = 0.02) were associated with the use of neuromuscular blockade.

**Fig. 4:**
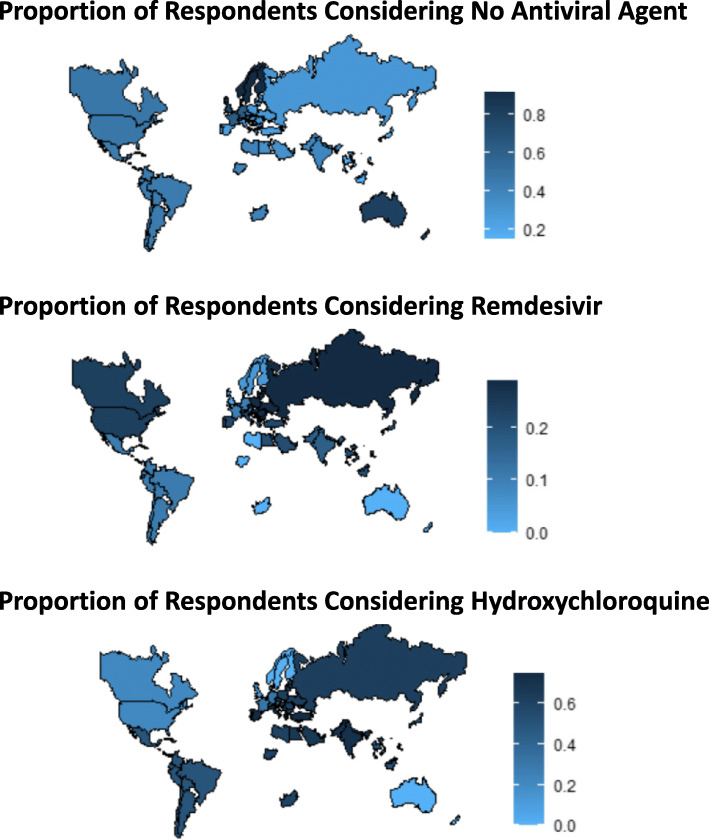
World map displaying practice variations across regions regarding the use of antiviral agents

Antibiotic prescribing was routine for all patients in 44.2% of the respondents and biomarker-guided in 36.5%, without significant variation across regions. Routine antibiotics were more frequently used by respondents working in university-affiliated hospitals (48.3% vs. 40.9%, *P* = 0.03) and those living in large cities (49.3% vs. 40.2%, *P* = 0.01). Biomarker-guided antibiotic therapy was less frequent in large cities (47.3% vs. 57.4%, *P* = 0.007). Regarding antiviral therapy, 48.9% reported not prescribing antivirals, 42.6% were giving hydroxychloroquine, 17% lopinavir-ritonavir, and 15% remdesivir. Figure 4 displays significant variation in antiviral prescriptions across regions (*P* < 0.0001). Physicians not prescribing antivirals were older (47 [40–54] vs. 44 [37–51] years, *P* < 0.0001), and more frequently men (55.4% vs. 39.9%, *P* < 0.0001). Conversely, those prescribing hydroxychloroquine were younger (43 [37–50] vs. 47 [40–54] years, *P* < 0.0001), and more frequently women (41.7% vs. 28.2%, *P* < 0.0001). There was significant variation in the use of interleukin-6 (IL-6)/IL-1 blockade or of corticosteroids across countries (*P* < 0.0001 for both tests). Other collected variables were not associated with the use of anti-inflammatory drugs.

## Discussion

This survey highlights important practice variations in the management of severe COVID-19 patients, including differences at regional and individual levels. This illustrates that neither IDSA nor Surviving Sepsis Guidelines did recommend any of these treatments, but instead encouraged inclusion of patients into trials [1, 4, 5]. Since the publication of these guidelines, no more evidence has been made available to ascertain that these specific COVID-19 therapies should be included in the standard of care. Learning from this heterogeneity will not only raise hypothesis on optimal patient’s management, but also serves as a tool to suggest personalized management for each clinical phenotype [6, 7].

This study has several limitations. First, the study suffers from a nonresponse bias of 80%. Second, even though only physicians have responded, we cannot ascertain that all of them had the clinical expertise and the experience of managing COVID-19 patients. Last, questions about specific treatments did not take into account the fact that the level of evidence has changed over time.

## Conclusion

As no management guidelines have allowed to guide practices for the COVID-19 pandemic, heterogeneous behaviors are reported. Large outcome studies based on multinational registries are warranted.

## Data Availability

Fully available upon request.
